# Association Between Superoxide Dismutase Isoenzyme Gene Expression and Total Antioxidant Status in Patients with an End-Stage Renal Disease

**DOI:** 10.4274/balkanmedj.2018.0170

**Published:** 2018-11-15

**Authors:** Ana Ninić, Miron Sopić, Jelena Munjas, Vesna Spasojević-Kalimanovska, Jelena Kotur-Stevuljević, Nataša Bogavac-Stanojević, Jasmina Ivanišević, Sanja Simić-Ogrizović, Milica Kravljača, Zorana Jelić-Ivanović

**Affiliations:** 1 Department for Medical Biochemistry, University of Belgrade, Faculty of Pharmacy, Belgrade, Serbia; 2 Clinic of Nephrology, Clinical Centre of Serbia, Belgrade, Serbia; 3 University of Belgrade, Faculty of Medicine, Belgrade, Serbia

**Keywords:** Antioxidants, end stage renal disease, ribonucleic acid, superoxide dismutase

## Abstract

**Background:**

Chronic renal failure, particularly end-stage renal disease, is a serious health problem associated with a high mortality rate. Uremic syndrome leads to increased oxidative stress, inflammation, and dyslipidemia.

**Aims:**

To examine superoxide dismutase isoenzyme gene expression in peripheral blood mononuclear cells of patients on hemodialysis and to determine the associations between superoxide dismutase isoenzyme gene expression, oxidative stress, and non-enzymatic antioxidative protection.

**Study Design:**

Case control study.

**Methods:**

This study included 33 patients on hemodialysis (age, 55.33±15.31 years old) and 33 apparently healthy controls (age, 45.37±8.92 years old). Superoxide dismutase isoenzyme messenger ribonucleic acid levels were determined by real-time polymerase chain reaction. General biochemical parameters, high sensitivity C-reactive protein, total antioxidant status, thiobarbituric acid-reactive substances, and the superoxide anion radical were also determined.

**Results:**

Normalized Cu/Zn superoxide dismutase and Mn superoxide dismutase messenger ribonucleic acid levels were significantly higher in patients than controls (p<0.001 and p=0.011). A significant negative correlation was detected between normalized Cu/Zn superoxide dismutase messenger ribonucleic acid levels and total protein, total cholesterol, high-density lipoprotein cholesterol, low-density lipoprotein cholesterol, and total antioxidant status. Normalized Mn superoxide dismutase messenger ribonucleic acid levels were negatively correlated with total protein and total antioxidant status. A multiple regression analysis revealed independent associations between total antioxidant status and normalized Cu/Zn superoxide dismutase (p=0.038) and between total antioxidant status and normalized Mn superoxide dismutase messenger ribonucleic acid levels (p=0.038 and p=0.018, respectively).

**Conclusion:**

The superoxide dismutase isoenzyme gene is expressed at a higher rate in patients with end-stage renal failure, probably due to increased oxidative stress and attenuated antioxidative defense. The plasma total antioxidant status is an independent predictor of normalized superoxide dismutase isoenzyme messenger ribonucleic acid levels.

Chronic kidney disease leads to the development of progressive and severe atherosclerosis, ischemic vascular disease, and cardiovascular death (1). The terminal stage of chronic kidney disease, classified as kidney failure, is treated using renal function replacement (hemodialysis, peritoneal dialysis, or kidney transplantation) (2,3). During hemodialysis, leukocytes are activated on the dialyzer membrane surface resulting in an inflammatory reaction and the generation of reactive oxygen species (ROS) (4), which impairs the function of tissues and cells, including leukocytes themselves. In addition, uremic toxins present at high concentrations in patients undergoing hemodialysis give rise to pro-inflammatory and pro-oxidant states (5). Uncontrolled ROS production that overwhelms antioxidant production and disrupts the homeostatic balance between oxidants and antioxidants is called oxidative stress. This stressed condition damages cellular components, proteins, lipids, and DNA and, ultimately, cell integrity (6). In contrast to their unfavorable effects, ROS also participate in modulating antioxidant gene expression and cellular signaling pathways (7,8). By activating redox-sensitive transcription factors, ROS enhance gene expression of Cu/Zn superoxide dismutase (SOD), Mn SOD, glutathione peroxidase (GPx), and catalase in epithelial cells and leucocytes (8,9). The superoxide anion radical (O_2_.-) is an extremely reactive substrate of SOD isoenzymes. If not scavenged properly, it causes lipid peroxidation (9). Malondialdehyde is a component of thiobarbiturate acid-reactive substances (TBARS) and the final product of lipid peroxidation. Malondialdehyde is an indicator of lipid oxidative damage in membranes of patients undergoing hemodialysis (10). In addition to enzymatic antioxidant protection comprised of SODs, GPx, and catalase, non-enzymatic antioxidant protection plays a very important role neutralizing ROS in cells and blood. Total antioxidant status (TAS) is a term that encompasses all non-enzymatic antioxidant molecules in the blood, such as proteins, urea, creatinine, bilirubin, uric acid, vitamin C, vitamin E, reduced glutathione, lipoic acid, β-carotene, and others (11). The antioxidant effects of these molecules are additive and they exert a significant role scavenging ROS in patients undergoing hemodialysis. As a consequence, TAS decreases, not only because of its consumption but also due to loss through the dialysis membrane. The aim of this study was to determine the influence of oxidative stress, antioxidative defense, and inflammatory states in the blood on SOD isoenzyme gene expression in peripheral blood mononuclear cells of patients undergoing hemodialysis.

## MATERIALS AND METHODS

Thirty-three patients (19 males and 14 females; mean age, 55 years) with chronic kidney disease receiving hemodialysis were included in the study. The diagnoses were as follows: six patients had chronic glomerulonephritis, six had nephroangiosclerosis, four had chronic renal insufficiency, three had uric acid nephropathies, three had Good-Pasture syndrome, two had renal calculosis, two had vesicoureteral reflux, one had hypertension, one had atrophia renis segmentalis, one had lupus nephritis, one had endemic nephropathy, one had chronic pyelonephritis, one had renal tuberculosis, and one had renal vasculitis. Because their kidneys were unable to perform physiological roles, hemodialysis was substituted. Cessation of basic kidney functions and the hemodialysis process itself increases oxidative stress (4,5), and, accordingly, all patients were analyzed together. The patients had been on hemodialysis ranging from 3 to 248 days. All patients undergoing hemodialysis were recruited from the nephrology clinic.

The control group was comprised of 33 healthy subjects (15 males and 18 females; mean age, 45 years). Inclusion criteria for the control group were the absence of a family history of cardiovascular disease, absence of hypertension (systolic blood pressure <140 mm Hg and/or diastolic blood pressure <90 mm Hg), (12) and/or the absence of any antihypertensive therapy, a favorable lipid profile according to Adult Treatment Panel III guidelines (13), and glucose <6.1 mmol/L. All participants were informed about the goals, procedures, and risks of participating in the study. They gave their consent for voluntary participation. The local Ethics Committee approved this study protocol. This research was carried out in compliance with the Declaration of Helsinki. After a 12 hour overnight fast, three blood samples were taken from the cubital vein using two Vacutainer systems with ethylenediaminetetraacetic acid as the anticoagulant to extract plasma and peripheral blood mononuclear cells and one Vacutainer had a serum separator gel for serum. Plasma and serum were collected by centrifugation for 10 min at 3.000 rpm, divided into aliquots, and stored at -80 °C until analysis, except for the determination of plasma O_2_.- which was performed immediately. Peripheral blood mononuclear cells were suspended in 1 mL of TRIzol^TM^ (Invitrogen Life Technologies, Foster City, CA, USA) reagent and frozen at -80 °C until total RNA isolation. Glucose, total cholesterol, high-density lipoprotein cholesterol (HDL-c), low-density lipoprotein cholesterol (LDL-c), triglycerides, total protein, urea, and creatinine were measured by routine enzymatic methods in plasma using the ILab 300+ analyzer (Instrumentation Laboratory, Milan, Italy) and Randox Laboratories reagents (Ardmore, UK). High sensitivity C-reactive protein (hsCRP) was determined by an immunoturbidimetric method using a COBAS^®^ c6000-Roche Diagnostics (Roche, Mannheim, Germany) analyzer.

Thiobarbituric acid-reacting substances (TBARS) were measured as the quantity of malondialdehyde-TBA 1:2 adducts spectrophotometrically at 535 nm (14). The rate of nitroblue tetrazolium reduction was used to measure O_2_.- (15). TAS was measured based on decolorization of the 2.2’-azinobis (3-ethylbenzothiazoline-6-sulfonic acid) radical cation (16). Total RNA was isolated using TRIzol^TM^ reagent according to an optimized procedure (17,18). The concentration, purity, and integrity of the isolated total RNA were determined by spectrophotometric analysis and native 1% agarose gel electrophoresis. Reverse transcription (complementary DNA synthesis) and real-time polymerase chain reaction (PCR) experiments were performed on a 7500 Real-Time PCR System (Applied Biosystems, Foster City, CA, USA). Cu/Zn SOD and Mn SOD gene expression [messenger RNA (mRNA)] were measured using TaqMan^TM^ Gene Expression Assays (primer sequences available upon request) according to the manufacturer’s instructions (Applied Biosystems). Gene expression data were calculated as follows: Normalized Cu/Zn SOD mRNA=Cu/Zn SOD mRNA/ β-actin mRNA; normalized Mn SOD mRNA=Mn SOD mRNA/β-actin mRNA. Negative controls for reverse transcription (without reverse transcriptase) and for real-time PCR (no complementary DNA) were included in all experiments.

### Statistical analysis

The Shapiro-Wilk test was used to examine the data distributions of the examined variables. Data are shown as arithmetic mean ± standard deviation for normally distributed variables. Data that achieved a Gaussian distribution after logarithmic transformation are presented as geometric mean [95% confidence intervals (CIs)] (19). Skewed data are presented as medians (interquartile range). Normal and log-normal continuous variables were compared with Student’s t-test. Skewed data were compared with the Mann-Whitney U test. Categorical variables are indicated as absolute frequencies and were compared using the chi-square test to prepare contingency tables. Pearson’s correlation analysis was used to estimate correlations between the parameters in the tested populations. Data from the correlation analysis are presented as coefficients of correlation (r). If probability values (p) for r were <0.05, those variables were further tested in a multiple linear regression analysis. A multiple regression analysis was performed to estimate the independent contribution of clinical markers on Cu/Zn SOD and Mn SOD gene expression. Data from the multiple regression models are presented as unstandardized coefficients (B), the 95% CIs, standardized coefficient (β), and the t-value. The F-ratio from the analysis of variance in the multiple regression analysis was used to determine whether the overall regression models provided a good data fit. Multicollinearity among the independent variables was also tested. Study power was calculated using the post-hoc Statistical Power Calculator for Multiple Regression (20). Statistical analyses were performed using SPSS version 22 software (SPSS Inc., Chicago, IL, USA). A p-value <0.05 was considered significant.

## RESULTS

The general characteristics of the study groups are presented in Table 1. As expected, significant differences were detected in all parameters between the groups except LDL-c concentration. Patients undergoing hemodialysis were older and had a lower body mass index than the controls. In addition, total protein and HDL-c concentrations were lower in the patients than controls. In contrast, urea, creatinine, total cholesterol, triglycerides, and hsCRP concentrations were significantly higher in the patients than the controls (Table 1). A disruption in oxidative stress and antioxidative defense was apparent in the patients: TBARS and O_2_.- were significantly higher in patients compared with the controls, and TAS was significantly lower in the patients than the controls. These results indicate that the patients were in a state of oxidative stress. Normalized Cu/Zn SOD and Mn SOD mRNAs were higher in the patients than in the controls (Table 2). Associations between the parameters and SOD isoenzyme gene expression were tested using Pearson’s correlation analysis. Normalized Cu/Zn SOD mRNA was positively correlated with urea, creatinine, and O_2_.-. Negative correlations were noted between normalized Cu/Zn SOD mRNA and total protein, total cholesterol, HDL-c, LDL-c, and TAS. Normalized Mn SOD mRNA was negatively correlated with TAS and total protein but positively correlated with creatinine (Table 3). We conducted a multiple linear regression analysis to identify the parameters independently associated with normalized Cu/Zn SOD and Mn SOD mRNAs. Independent variables that were correlated with normalized Cu/Zn SOD mRNA in the Pearson’s correlation analysis with a p<0.05 (Table 3) were grouped into model 1. These independent variables (total protein, creatinine, HDL-c, LDL-c, TAS, and O_2_.-) determined in serum significantly predicted normalized Cu/Zn SOD mRNA in peripheral blood mononuclear cells [F(6.48)=5.249, p<0.001)]. This result also demonstrated that model 1 was a good fit for the data. An adjusted R^2^ of 0.339 for model 1 demonstrated that 33.9% of the variation in the normalized Cu/Zn SOD mRNA in peripheral blood mononuclear cells was explained by model 1. Of all parameters, only TAS was independently associated with a decrease in normalized Cu/Zn SOD mRNA (β=-0.311, p=0.038) (Table 4). Urea and total cholesterol were excluded from model 1 due to multicollinearity. The independent variables that correlated with normalized Mn SOD mRNA in Pearson’s correlation analysis at p<0.05 (Table 3) were grouped into model 2. These independent variables (total protein, creatinine, and TAS) determined in serum significantly predicted normalized Mn SOD mRNA in peripheral blood mononuclear cells [F(3.52)=5.879, p=0.002]. Model 2 was also a good fit for the data. An adjusted R^2^ of 0.210 demonstrated that 21% of the variation in Mn SOD mRNA could be explained by model 2. As shown for model 1, only TAS was independently associated with a decrease in normalized Mn SOD mRNA (β=-0.374, p=0.018) (Table 5). The post-hoc calculated statistical power values for the study from the multiple linear regression analysis were 0.99 and 0.98 according to models 1 and 2, respectively.

## DISCUSSION

Many factors are involved in the pathogenesis of oxidative stress in patients with chronic kidney disease receiving hemodialysis. Oxidative stress in patients with chronic kidney disease occurs before patients begin hemodialysis (5,21). Besides metabolic and functional organ disorders that occur in chronic kidney disease, hemodialysis itself contributes to enhance oxidative stress, through production of ROS (especially O_2_.-), such as when leukocytes contact the hemodialysis membrane. A decrease in blood antioxidants, which occurs because of their consumption during ROS neutralization and loss through the dialyzer membrane, additionally contribute to increase oxidative stress (10,22). According to our results, the patients were in a state of oxidative stress (Table 2). TBARS and O_2_.- were significantly higher and TAS was significantly lower in patients than in controls (Table 2). Furthermore, TAS was negatively correlated with TBARS and O_2_.- (r=-0.328, p=0.008 and r=-0.515, p<0.001, respectively). TBARS and O_2_.- were positively correlated (r=0.328, p=0.008) (data not shown). Malondialdehyde, a component of TBARS, is the ultimate product of lipid peroxidation and an oxidative stress marker (10). An increase in malondialdehyde concentration indicates higher ROS production. Reduced elimination of ROS can be a consequence of higher TBARS production (10,23). In addition to very high reactivity, O_2_.- inhibits nitric oxide-dependent vasodilation, which could impair cardiovascular function leading to a high mortality rate in patients undergoing hemodialysis (24). TAS, which includes all non-enzymatic components in plasma (11), is an important parameter to assess the capacity of a biological system to prevent oxidative stress. As shown in Table 1, urea and creatinine were significantly higher in patients than in controls (p<0.001, for both), and total protein was significantly higher in controls than in patients (p=0.007). As most of TAS is comprised of protein (>50%) (11,16), it was expected that TAS would be lower in patients than in controls. Previous studies have shown that TAS is lower in patients before and after hemodialysis compared with controls (25,26). According to a study by Zargari and Sedighi (25) and in line with ours, a pronounced increase in TBARS and O_2_.-, a decrease in TAS, and their significant negative correlation indicate a significant burden and decrease in non-enzymatic antioxidant protection due to chronic exposure to ROS. Activated leukocytes provide a link between oxidative stress and inflammation (5,10). In Uremic syndrome, chronic activation of the immune system leads to continuous low-grade inflammation characterized by increased cytokines (tumor necrosis factor-α, interleukin-1, and interleukin-6) and CRP in the blood. The patients in our study were in a state of oxidative stress and had an hsCRP concentration of 2.46 mg/dL (95% CI 1.30-4.60 mg/dL), indicating low-grade inflammation. In addition, CRP is a potent stimulator of antioxidant enzyme gene expression in leukocytes. One of the main objectives of our study was to examine gene expression of SOD isoenzymes in peripheral blood mononuclear cells from patients receiving hemodialysis, to compare it with that from controls, and to determine which oxidative stress status parameters regulated expression of their genes. Leukocytes are surrogate cells for an antioxidant enzyme gene expression analysis, not only because they are a source of ROS, but also because they are the target sites of the effects of ROS (4,9,27). During contact with the hemodialysis membrane, activated leukocytes release ROS and cytokines that further affect the leukocytes themselves and other cells with which they come into contact with. These events induce antioxidant enzyme gene expression (27,28). These results confirmed previous findings. Normalized Cu/Zn SOD and Mn SOD mRNAs were significantly higher in patients, who were in oxidative stress and low-grade inflammation states, than in controls (Table 2). Only a few studies have investigated SOD isoenzyme gene expression in leukocytes from patients undergoing hemodialysis (29,30) with conclusions similar to ours. However, the main cause for the induction of SOD genes remains unknown. Some authors (29,30) have hypothesized that chronic oxidative stress and inflammation in Uremic syndrome contributes to increase induction of SOD isoenzyme genes due to high CRP and O_2_.- as positive modulators of anti-oxidative enzyme transcriptional processes in cells. O_2_.- was positively correlated only with normalized Cu/Zn SOD mRNA (Table 3). Moreover, we were unable to establish any significant correlation between normalized SOD isoenzyme mRNAs and hsCRP. Although patients on hemodialysis were older than the controls (Table 1), we assumed no significant impact of age on normalized Cu/Zn SOD or Mn SOD mRNAs because there was no significant correlation between normalized SOD isoenzyme mRNAs and age (Table 3). In addition, no significant differences in normalized SOD isoenzyme mRNAs were observed when compared between age tertiles separately in patients and controls (data not presented).

According to our results, elevated Cu/Zn SOD and Mn SOD gene expression in peripheral blood mononuclear cells from the patients (Table 2) probably indicates a defense mechanism against oxidative stress and the consumption of plasma antioxidants. Schettler et al. (27,30) concluded similarly. They assumed that induction of SOD isoenzyme gene expression occurs in other organs as well, but there is insufficient evidence for this claim in the literature. Furthermore, our results show significant associations between plasma antioxidants and Cu/Zn SOD and Mn SOD gene expression in peripheral blood mononuclear cells. Normalized Cu/Zn SOD mRNA was negatively correlated with TAS but positively correlated with O_2_.-. Normalized Mn SOD mRNA was negatively correlated with TAS (Table 3). As TAS decreased, Cu/Zn SOD and Mn SOD mRNAs increased, which acted as an adaptive mechanism to decrease antioxidants and increase oxidative stress. TAS was an independent predictor of normalized Cu/Zn SOD and Mn SOD mRNAs when tested by multiple linear regression analysis (β=-0.311, p=0.038 and β=-0.374, p=0.018, respectively) (Table 4, 5). Model 1 significantly predicted normalized Cu/Zn SOD mRNA in peripheral blood mononuclear cells [F(6.48)=5.249, p<0.001] and explained 33.9% of the variation in its levels. Model 2 significantly predicted normalized Mn SOD mRNA [F(3.52)=5.879, p=0.002] and explained 21% of the variation in its levels. O_2_.- in the blood failed to independently predict normalized Cu/Zn SOD mRNA (Table 4). This is the first study to demonstrate independent associations between SOD isoenzyme gene expression in peripheral blood mononuclear cells and TAS in blood. Two limitations of this study should be mentioned. First, we only had access to a small number of controls and patients; hence, our conclusions need to be confirmed in a larger study. Second, SOD isoenzyme activities and concentrations were not determined. As variations in gene expression do not necessarily reflect protein abundance and activity, this limitation remains to be explored. Patients undergoing hemodialysis with chronic kidney disease were in a state of increased oxidative stress. Higher O_2_.- combined with exhaustion of non-enzymatic antioxidative defenses (as shown by the decreased TAS values) are likely to be key inducers of Cu/Zn SOD and Mn SOD genes in peripheral blood mononuclear cells. Only TAS was an independent predictor of SOD isoenzyme gene expression in peripheral blood mononuclear cells.

## Figures and Tables

**Table 1:**
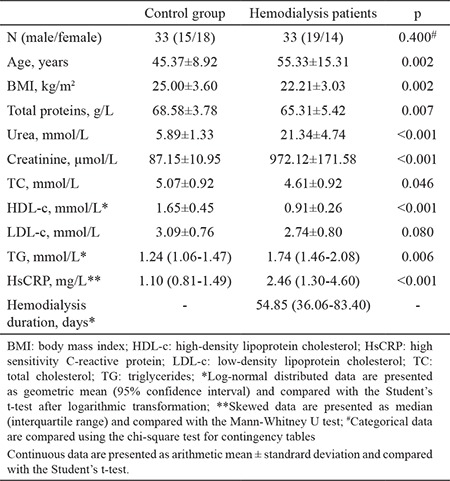
Demographic and laboratory parameters in the tested populations

**Table 2:**
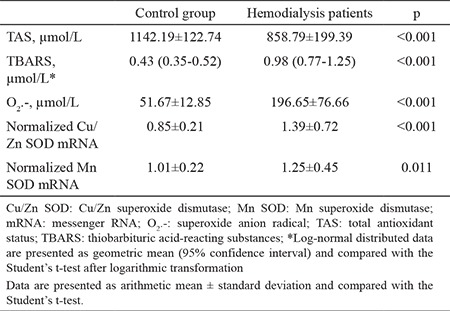
Oxidative stress status and antioxidative defense parameters in the tested populations

**Table 3:**
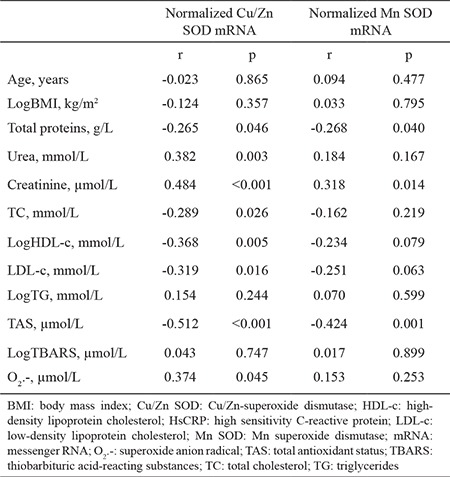
Bivariate Pearson’s correlation analysis between Cu/Zn SOD and Mn SOD messenger RNA s and other clinical parameters

**Table 4:**
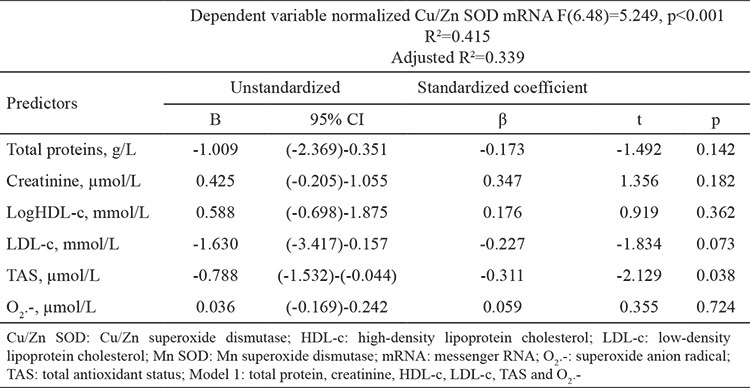
Multiple regression analysis for independent association of examined parameters with Cu/Zn SOD gene expression levels

**Table 5:**
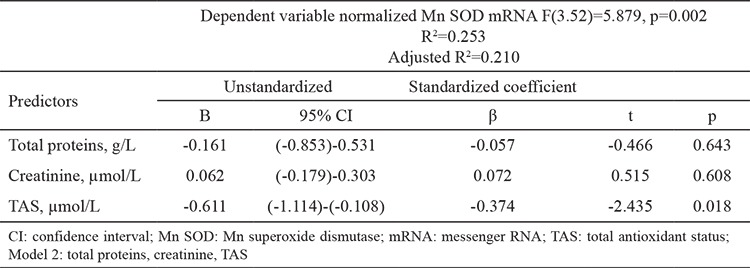
Multiple regression analysis for the independent associations between the examined parameters and Mn superoxide dismutase gene expression levels
